# Pathogenic cytokines in thrombotic microangiopathies: molecular insights and therapeutic targets

**DOI:** 10.1186/s10020-025-01331-1

**Published:** 2025-10-24

**Authors:** Emmanuel Ifeanyi Obeagu

**Affiliations:** 1 Department of Biomedical and Laboratory Science, Africa University, Mutare, Zimbabwe; 2 Department of Medical Laboratory Science, Kampala International University, Ishaka, Uganda

**Keywords:** Cytokines, Thrombotic microangiopathies, Endothelial dysfunction, Inflammation, Targeted therapy

## Abstract

Thrombotic microangiopathies (TMAs) are a heterogeneous group of disorders characterized by endothelial damage, microvascular thrombosis, thrombocytopenia, and microangiopathic hemolytic anemia. While the initiating triggers may differ—ranging from infections and autoimmune diseases to genetic complement dysregulation—a unifying pathophysiological feature is injury to the vascular endothelium. Recent advances have highlighted the critical role of pro-inflammatory cytokines in mediating endothelial dysfunction, contributing to both the initiation and propagation of thrombotic events in TMAs. Cytokines such as tumor necrosis factor-alpha (TNF-α), interleukin-6 (IL-6), and interleukin-1β (IL-1β) have been implicated in promoting endothelial activation, increased permeability, leukocyte adhesion, and procoagulant changes. These effects contribute to the loss of vascular integrity and the formation of microthrombi. Moreover, cytokine-mediated inflammation appears to be a common feature across various TMA subtypes, including Shiga toxin-associated hemolytic uremic syndrome (HUS), atypical HUS, thrombotic thrombocytopenic purpura (TTP), and secondary TMAs. The intensity and profile of cytokine involvement may vary, but their pathological influence on endothelial health remains a shared mechanism.

## Introduction

Thrombotic microangiopathies (TMAs) represent a diverse group of disorders unified by a shared pathological hallmark: microvascular thrombosis leading to organ injury. Clinically, TMAs are characterized by microangiopathic hemolytic anemia, thrombocytopenia, and end-organ dysfunction, particularly affecting the kidneys and central nervous system. Common syndromes under the TMA umbrella include thrombotic thrombocytopenic purpura (TTP), typical and atypical hemolytic uremic syndrome (HUS), and secondary TMAs associated with malignancies, autoimmune diseases, infections, and certain medications (Chang 2022; Blasco et al. 2022). While the etiologies of TMAs vary, one central mechanism drives disease progression across all subtypes—endothelial cell injury. Damage to the vascular endothelium disrupts the antithrombotic barrier function of the microvasculature, initiating a cascade of events that culminate in platelet aggregation, thrombus formation, and vascular occlusion. Historically, mechanical shear stress and complement dysregulation have been emphasized in the pathogenesis of TMAs. However, recent advances in immunopathology have highlighted the pivotal role of inflammation—particularly the actions of pro-inflammatory cytokines—in endothelial activation and dysfunction (Vorobev et al. 2024; Mazzierli et al. 2023). Cytokines are small soluble proteins that act as mediators of immune and inflammatory responses. In the context of TMA, cytokines such as tumor necrosis factor-alpha (TNF-α), interleukin-6 (IL-6), and interleukin-1β (IL-1β) have been implicated in promoting endothelial injury through various mechanisms including increased permeability, apoptosis, leukocyte adhesion, and upregulation of procoagulant molecules. These inflammatory mediators not only initiate vascular injury but also amplify and sustain the microthrombotic process, particularly in immune-mediated or complement-activated TMAs (Minoia et al. 2021).

In typical HUS, often associated with Shiga toxin-producing *Escherichia coli*, cytokine release is a downstream effect of toxin-induced endothelial damage. The toxin sensitizes endothelial cells to TNF-α and other cytokines, enhancing the inflammatory response and contributing to microvascular thrombosis. In atypical HUS, complement dysregulation triggers cytokine production, which exacerbates endothelial dysfunction. Similarly, in TTP, although the primary defect lies in ADAMTS13 deficiency, cytokines are involved in endothelial activation and may worsen clinical outcomes (Paramo 2021; Blasco et al. 2021). Secondary TMAs, such as those seen in autoimmune diseases like systemic lupus erythematosus or during pregnancy (e.g., HELLP syndrome), are often driven by systemic inflammation. In these settings, cytokine profiles are often markedly elevated, correlating with disease severity and organ damage. The widespread activation of endothelial cells by cytokines creates a systemic prothrombotic environment, further blurring the lines between inflammatory and thrombotic pathways (Java and Kim 2023; Liles 2023). The growing body of evidence supporting the role of cytokines in TMA pathophysiology has prompted investigations into cytokine-targeted therapies. Biologic agents that inhibit IL-6, IL-1, or TNF-α are already used in other inflammatory and autoimmune conditions and are being explored in TMA syndromes. Furthermore, treatments that modulate complement activity, such as eculizumab, may have indirect effects on cytokine release and inflammatory endothelial responses (Filippone et al. 2021).

### Aim

The aim of this review is to comprehensively examine the role of pathogenic cytokines in the development and progression of thrombotic microangiopathies (TMAs), with a focus on their contribution to endothelial dysfunction and microvascular thrombosis.

### Clinical trial number

Not applicable as this a narrative review.

### Classification and pathogenesis of thrombotic microangiopathies

Thrombotic microangiopathies (TMAs) encompass a spectrum of clinical syndromes characterized by small vessel thrombosis, leading to organ dysfunction, particularly of the kidneys and central nervous system. The term TMA is descriptive, not diagnostic, as it refers to the histopathologic finding of endothelial swelling, subendothelial expansion, and platelet-rich thrombi within arterioles and capillaries. The classification of TMAs has evolved over time, now largely guided by underlying etiology and pathophysiologic mechanisms (Kim 2022; Fakhouri and Frémeaux-Bacchi 2021). TMAs are broadly classified into primary and secondary forms. Among the primary TMAs, thrombotic thrombocytopenic purpura (TTP) and hemolytic uremic syndrome (HUS) are the most recognized entities. TTP is defined by a severe deficiency of ADAMTS13, a metalloprotease responsible for cleaving ultra-large von Willebrand factor (vWF) multimers. Without adequate ADAMTS13 activity, these multimers accumulate and promote spontaneous platelet aggregation within the microcirculation. Clinically, TTP is characterized by thrombocytopenia, microangiopathic hemolytic anemia, and varying degrees of neurological and renal involvement (McFarlane et al. 2021; Palma et al. 2021).

HUS, on the other hand, is classically divided into typical (Shiga toxin-associated) and atypical forms. Typical HUS is primarily triggered by Shiga toxin-producing *Escherichia coli* (STEC), most commonly affecting children following a diarrheal illness. The Shiga toxin binds to receptors on glomerular endothelial cells, inducing apoptosis and triggering cytokine release, which culminates in thrombotic injury. In contrast, atypical HUS (aHUS) is driven by dysregulation of the alternative complement pathway. Mutations or autoantibodies targeting regulatory proteins like factor H, factor I, or membrane cofactor protein lead to uncontrolled complement activation, resulting in direct endothelial injury, recruitment of inflammatory mediators, and propagation of thrombosis (Mazzierli et al. 2023). Secondary TMAs develop in the context of other systemic insults. These include conditions such as pregnancy (e.g., HELLP syndrome), autoimmune diseases (notably systemic lupus erythematosus and antiphospholipid syndrome), malignant hypertension, infections, bone marrow transplantation, and certain drugs (e.g., calcineurin inhibitors or chemotherapeutic agents). The pathogenesis of secondary TMAs is multifactorial, often involving a combination of mechanical stress, immune dysregulation, and proinflammatory cytokine activity that converge on endothelial injury and microvascular thrombosis (Vorobev et al. 2024; Doorn et al. 2025).

Despite the heterogeneity in cause, endothelial dysfunction remains the central feature in all TMAs. The activated or injured endothelium expresses adhesion molecules, releases procoagulant factors, and becomes permeable to circulating cells and proteins. This prothrombotic transformation of the vascular lining not only initiates platelet aggregation but also amplifies the inflammatory response, creating a vicious cycle of thrombosis and tissue ischemia. Cytokines, especially TNF-α, IL-6, and IL-1β, are increasingly recognized as both markers and mediators in this process, contributing to the perpetuation of vascular injury across different TMA subtypes (Mubarak et al. 2024; Henry et al. 2021). This evolving understanding of the classification and pathogenesis of TMAs underscores the importance of accurate diagnosis and tailored treatment strategies. Recognition of the molecular pathways involved, including the roles of ADAMTS13, complement, and cytokines, is essential for guiding effective therapy and improving outcomes in patients with these potentially life-threatening disorders (Leisring et al. 2024).

The term"complement-mediated thrombotic microangiopathy (TMA)"has emerged as a more precise and encompassing designation for a group of TMAs primarily driven by dysregulation of the complement system, particularly the alternative pathway. Historically, atypical hemolytic uremic syndrome (aHUS) was the clinical term used to distinguish non-Shiga toxin-associated HUS from typical, infection-related forms. However, advances in molecular and genetic understanding have revealed that many cases of aHUS are underpinned by genetic mutations or autoantibodies affecting complement regulatory proteins such as factor H (CFH), factor I (CFI), membrane cofactor protein (CD46), C3, and factor B (CFB), leading to uncontrolled complement activation and endothelial injury. Additionally, the presence of autoantibodies against complement factor H, often in the context of CFHR1/3 deletions, further implicates acquired complement dysregulation in disease pathogenesis. As such, the term “complement-mediated TMA” is now increasingly used to reflect this shared pathogenic mechanism rather than relying solely on clinical exclusion. This shift in terminology emphasizes the central role of complement overactivation as a unifying driver of microangiopathic injury and helps delineate this subset from other TMAs with similar clinical presentations but distinct etiologies, such as TTP or secondary TMAs. Recognizing this category is crucial, as it directly informs diagnostic testing for complement abnormalities and the use of targeted therapies such as eculizumab and ravulizumab, which inhibit terminal complement activation (Noris and Remuzzi 2009, 2013; Fakhouri et al. 2017).

### Role of cytokines in endothelial dysfunction

Endothelial dysfunction lies at the heart of thrombotic microangiopathies (TMAs), serving as both an initiator and a perpetuator of microvascular injury. The vascular endothelium, which normally functions as an anti-thrombotic, anti-inflammatory barrier, becomes pro-thrombotic and pro-inflammatory under pathological conditions. One of the most critical mediators of this transition is the dysregulated release of cytokines—small signaling proteins that orchestrate immune responses, inflammation, and intercellular communication. In TMAs, cytokine-driven endothelial activation transforms the vascular lining from a protective surface into one that facilitates platelet adhesion, leukocyte infiltration, and thrombosis (Noris and Remuzzi 2009, 2013). Among the most well-studied cytokines implicated in endothelial dysfunction are tumor necrosis factor-alpha (TNF-α)**,** interleukin-6 (IL-6), and interleukin-1 beta (IL-1β). TNF-α, produced primarily by activated macrophages and monocytes, induces a cascade of detrimental effects on endothelial cells. It upregulates adhesion molecules such as ICAM-1 and VCAM-1, promoting leukocyte adherence and transmigration. TNF-α also stimulates the release of tissue factor and suppresses thrombomodulin expression, tipping the hemostatic balance toward coagulation. Furthermore, TNF-α can induce endothelial apoptosis, directly compromising vascular integrity and promoting exposure of the subendothelial matrix to circulating platelets (Kei et al. 2023; Liu et al. 2025).

IL-6, another key cytokine elevated in various forms of TMA, plays a multifaceted role in vascular pathology. It drives hepatic production of acute-phase reactants such as fibrinogen and C-reactive protein, both of which contribute to a prothrombotic state. Within the endothelium, IL-6 increases permeability and modulates vascular tone, creating conditions favorable for both thrombus formation and organ injury. Elevated IL-6 levels have been associated with disease severity in TMAs, and therapies targeting IL-6 signaling, such as tocilizumab, are being explored in related inflammatory and thrombotic conditions (Zhang and Dhalla 2024; Malhab et al. 2021; Koper-Lenkiewicz et al. 2022). IL-1β contributes further to endothelial injury by enhancing vascular permeability, promoting leukocyte chemotaxis, and stimulating the release of additional cytokines and reactive oxygen species. Like TNF-α and IL-6, IL-1β induces expression of adhesion molecules and tissue factor, reinforcing the pro-inflammatory and pro-coagulant phenotype of activated endothelial cells. The combined effect of these cytokines results in a loss of endothelial homeostasis and the formation of microvascular thrombi that define the clinical picture of TMAs (Vorobev et al. 2024; Abdelkader et al. 2023). Cytokine activity is not limited to a single pathway but is part of a broader inflammatory network that synergistically amplifies endothelial damage. The reciprocal activation of immune cells and endothelial cells creates a self-perpetuating loop of cytokine release and vascular injury. In complement-mediated TMAs, such as atypical HUS, complement activation further exacerbates this process by inducing additional cytokine release and promoting endothelial cell lysis. In TTP, although ADAMTS13 deficiency is the central defect, elevated cytokines may enhance von Willebrand factor (vWF) release and leukocyte-endothelium interactions, compounding microthrombus formation (Guo et al. 2023; Watanabe-Kusunoki et al. 2024).

### Cytokine-centric pathways in specific TMA subtypes

Although thrombotic microangiopathies (TMAs) share a common pathological feature—microvascular thrombosis driven by endothelial injury—the underlying mechanisms vary across different clinical subtypes. Despite their distinct etiologies, many forms of TMA converge on a cytokine-mediated inflammatory response that contributes significantly to disease progression. The degree and nature of cytokine involvement vary depending on the trigger, but in each subtype, these mediators play a critical role in amplifying endothelial dysfunction and promoting thrombosis (Al-Tamimi et al. 2022). In Shiga toxin-associated hemolytic uremic syndrome (STEC-HUS), the initiating insult arises from Shiga toxin produced by enterohemorrhagic *Escherichia coli*. Once absorbed into the bloodstream, the toxin binds to globotriaosylceramide (Gb3) receptors, which are abundantly expressed on renal glomerular endothelial cells. Binding leads to ribosomal inactivation, apoptosis, and heightened susceptibility to pro-inflammatory stimuli. Shiga toxin also stimulates the release of cytokines such as TNF-α and IL-1β from monocytes and endothelial cells. These cytokines not only exacerbate vascular inflammation and thrombosis but also upregulate Gb3 expression, creating a feed-forward loop that intensifies endothelial injury and microvascular occlusion (Table 1) (Timmermans and Paassen 2021).

**Table 1:** Cytokine alterations in specific TMA subtypes and corresponding model systems

TMA Subtype	Cytokine	Alteration	Model System	Reference
TTP (Thrombotic Thrombocytopenic Purpura)	IL-6	↑ Elevated in acute episodes; correlates with severity	Clinical (patients)	Coppo et al., 2010 (PMID: 20130401)
	IL-8	↑ Associated with neutrophil activation and NETs	Clinical	Carmona-Rivera et al., 2022 (PMID: 26250777)
	TNF-α	↑ Implicated in ADAMTS13 suppression	In vitro, Clinical	Uemura et al. 2011 (PMID: 21994870)
STEC-HUS (Shiga toxin-associated HUS)	IL-6	↑ Induced by Shiga toxin; worsens renal injury	Murine, Clinical	Hunt et al., 2017 (PMID: 22518003)
	IL-1β	↑ Amplifies endothelial activation	Murine, In vitro	Keepers et al., 2020 (PMID: 17671662)
	TNF-α	↑ Enhances Gb3 receptor expression, toxin uptake	In vitro	(Obrig et al. 1993 ) (PMID: 8381385)
aHUS (Atypical HUS/Complement-mediated TMA)	IL-6	↑ Elevates endothelial permeability; may trigger complement activation	Clinical	Fakhouri et al., 2020 (PMID: 32203195)
	IL-10	↓ Low levels may impair anti-inflammatory control	Clinical	Nester et al., 2015 (PMID: 25938789)
	C5a (Anaphylatoxin)	↑ Strongly pro-inflammatory; recruits neutrophils	Murine, Clinical	Brocklebank et al., 2017 (PMID: 23470623)
Secondary TMA (e.g., lupus, pregnancy, malignancy)	IL-6	↑ Elevated in lupus-related TMA	Clinical	Ramgopal et al., 2021 (PMID: 28350170)
	IFN-γ	↑ In lupus-related endothelial injury	In vitro, Clinical	Timmermans and Paassen (Timmermans, et al., 2021) (PMID: 19176316)
	TNF-α	↑ Contributes to transplant-associated TMA	Clinical	Jodele et al., 2014 (PMID: 25077612)

In atypical HUS (aHUS), the pathogenesis is largely driven by uncontrolled activation of the alternative complement pathway, often due to genetic mutations or autoantibodies against regulatory proteins like factor H or factor I. Complement overactivation leads to the deposition of membrane attack complexes (MACs) on endothelial surfaces, triggering direct cytotoxicity. In response to this complement-mediated injury, endothelial cells and immune cells release pro-inflammatory cytokines such as IL-6, IL-8, and TNF-α. These cytokines further damage the endothelium, enhance leukocyte adhesion, and upregulate procoagulant factors, thereby compounding thrombotic risk. While complement inhibition with agents like eculizumab can halt the primary insult, residual inflammatory cytokine activity may still contribute to ongoing vascular injury, especially in delayed or partial responses (Riedl Khursigara et al. 2022). In thrombotic thrombocytopenic purpura (TTP), the hallmark is a severe deficiency of ADAMTS13, which is either congenital or acquired through autoantibody inhibition. The resulting accumulation of ultra-large von Willebrand factor (vWF) multimers promotes spontaneous platelet aggregation in the microcirculation. While this enzymatic defect is central, the disease course is often influenced by inflammation. Elevated levels of IL-6 and TNF-α have been observed during acute TTP episodes and are thought to contribute to endothelial activation and vWF release. These cytokines also enhance the expression of P-selectin and other adhesion molecules, increasing platelet and leukocyte interaction with the endothelium. Moreover, inflammation may lower ADAMTS13 activity further, suggesting a bidirectional relationship between cytokines and the primary molecular defect (Mandhair et al. 2024).

Secondary TMAs, including those associated with autoimmune diseases, malignancies, infections, or pregnancy, often involve a robust systemic inflammatory response. In systemic lupus erythematosus (SLE), for example, type I interferons and TNF-α contribute to endothelial cell activation and the formation of immune complexes that exacerbate vascular injury. In HELLP syndrome—a severe variant of preeclampsia—placental ischemia and oxidative stress drive the release of cytokines like IL-6 and IL-8, which promote platelet activation, endothelial dysfunction, and coagulation cascade activation. In transplant-associated TMA or drug-induced TMA, cytokines play a modulatory role by exacerbating endothelial stress imposed by immunosuppressants or chemotherapeutic agents (Zhou et al. 2025). Each of these TMA subtypes illustrates a unique cytokine signature that interacts with the underlying pathology to amplify endothelial damage and thrombotic risk. While cytokines are not the sole drivers of TMAs, their role as amplifiers of injury places them at the crossroads of inflammation and thrombosis. Recognizing these cytokine-centric pathways provides a deeper understanding of disease mechanisms and opens the door to targeted therapies that may modulate inflammation, preserve endothelial function, and improve clinical outcomes across the spectrum of TMA disorders (Tian et al. 2024).

### Endothelial heterogeneity and cytokine sensitivity in organ-specific TMA lesions

**Fig. 1:**
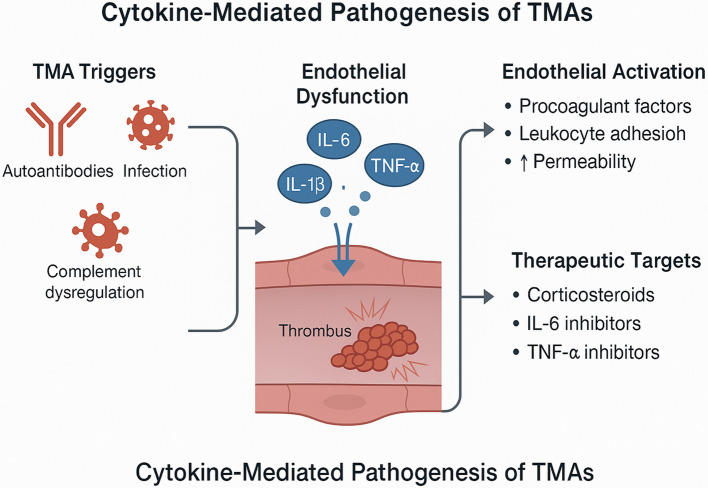
Cytokine-mediated pathogenesis of TMAs

The endothelium is not a uniform tissue but a highly specialized and heterogeneous layer of cells that varies significantly depending on the vascular bed and organ system. This heterogeneity is reflected in differences in morphology, gene expression profiles, surface receptors, and functional responses to inflammatory stimuli, including cytokines. These differences are critical to understanding the organotropism observed in TMAs, where lesions predominantly affect the kidney, brain, and gastrointestinal tract. In renal microvasculature, glomerular endothelial cells are uniquely fenestrated and have a dense glycocalyx, making them highly susceptible to complement activation and cytokine-induced injury. For example, proinflammatory cytokines such as TNF-α and IL-1β have been shown to upregulate adhesion molecules and increase vascular permeability in glomerular endothelial cells, predisposing to thrombus formation (Satchell and Braet 2009). In contrast, cerebral endothelial cells are part of the blood–brain barrier (BBB) and possess tight junctions that restrict molecular and cellular trafficking. These cells are relatively less permeable but respond to cytokines like IL-6 and IFN-γ by altering tight junction integrity and facilitating leukocyte adhesion, which may contribute to neurological manifestations in TTP and aHUS. Similarly, gastrointestinal endothelial cells have high expression of receptors for Shiga toxin (e.g., Gb3), which explains the severe colitis and hemorrhagic diarrhea often seen in STEC-HUS. Exposure to cytokines such as TNF-α further enhances Gb3 expression, increasing toxin uptake and endothelial injury in these vascular beds (Daneman and Prat 2015). This vascular bed–specific responsiveness to cytokines is influenced by both intrinsic genetic programming and local microenvironmental factors, including oxygen tension, mechanical stress, and the resident immune milieu. The differential expression of cytokine receptors, such as TNFR1, IL-6R, and type I interferon receptors, contributes to selective vulnerability and organ-specific endothelial activation in TMA (Fig. 1)(Obrig et al. 1993).

### The dual role of the endothelium: target and amplifier of cytokine-mediated injury in TMA

In the pathogenesis of thrombotic microangiopathies (TMAs), the vascular endothelium plays a dual role: it is both a primary target of injury and an active participant in amplifying the inflammatory response. Under physiological conditions, endothelial cells maintain vascular integrity, regulate hemostasis, and suppress immune activation. However, in TMA, this balance is disrupted by the convergence of complement overactivation, shear stress, and proinflammatory cytokines, leading to endothelial dysfunction and a prothrombotic state. Importantly, cytokines such as tumor necrosis factor-alpha (TNF-α), interleukin-1β (IL-1β), and interleukin-6 (IL-6) not only induce endothelial activation but also stimulate the endothelium itself to produce more cytokines and chemokines, thereby creating a self-reinforcing inflammatory loop. A pivotal study by Leligdowicz et al. demonstrated that endothelial cells exposed to plasma from patients with TMA exhibit a gene expression signature enriched for inflammatory mediators, including IL-6, IL-8, and MCP-1. This response was associated with increased vascular permeability, leukocyte adhesion, and thrombogenicity, hallmarks of TMA pathophysiology (Leligdowicz et al. 2018).

Moreover, endothelial cells, once activated, upregulate adhesion molecules such as ICAM-1, VCAM-1, and E-selectin, facilitating immune cell recruitment and further cytokine release. This feed-forward mechanism not only propagates vascular inflammation but also exacerbates endothelial injury, leading to microvascular thrombosis and organ dysfunction. Studies in both murine models and in vitro systems confirm that cytokine-stimulated endothelial cells can perpetuate inflammation via autocrine and paracrine loops (Looney et al. 2011). In addition, vascular bed-specific differences in endothelial cytokine production and receptor expression may contribute to organ tropism observed in TMA. For instance, glomerular endothelial cells are particularly responsive to IL-6 and complement-mediated injury, which may underlie the predominant renal involvement in atypical hemolytic uremic syndrome (aHUS) (Clark et al. 2007). Thus, targeting the endothelial-intrinsic cytokine response may represent a novel therapeutic strategy in TMA, especially in forms where cytokine-driven inflammation plays a prominent role, such as transplant-associated TMA and secondary TMAs associated with autoimmune disease or malignancy.

### Cytokine profiles across TMA subtypes: insights from clinical and experimental studies

A growing body of evidence supports the role of proinflammatory cytokines in the pathogenesis and progression of various thrombotic microangiopathy (TMA) subtypes. While complement dysregulation, ADAMTS13 deficiency, and mechanical or immune-mediated triggers are well-established contributors, cytokine-driven endothelial activation and dysfunction have emerged as common pathophysiological threads across different TMA contexts. Recent clinical and preclinical studies have provided valuable insights into distinct cytokine signatures associated with specific TMA subtypes, offering opportunities for both biomarker development and therapeutic intervention. In complement-mediated TMA (e.g., atypical hemolytic uremic syndrome, aHUS), elevated serum levels of IL-6, IL-1β, and TNF-α have been observed in both patients and murine models. These cytokines not only reflect the degree of endothelial inflammation but may also act upstream to amplify alternative pathway complement activation, creating a vicious cycle of microvascular injury. Additionally, IL-8 and MCP-1 have been implicated in leukocyte recruitment and glomerular infiltration in animal models of Ahus (George and Nester 2014).

In Shiga toxin-producing Escherichia coli (STEC)-HUS, human studies have demonstrated a prominent increase in IL-6, IL-8, and TNF-α, particularly during the acute hemolytic phase. Experimental models show that Shiga toxin enhances cytokine secretion by endothelial and intestinal epithelial cells, further increasing expression of Gb3 receptors, and potentiating toxin uptake and vascular damage. Thrombotic thrombocytopenic purpura (TTP), characterized by ADAMTS13 deficiency, is associated with immune dysregulation and endothelial stress. Elevated interferon-γ (IFN-γ), IL-10, and CXCL10 (IP-10) have been reported in plasma samples from acute TTP patients, indicating an active immune response involving both pro- and anti-inflammatory cytokines. Notably, these profiles differ from those seen in aHUS or STEC-HUS, underscoring disease-specific immune activation patterns (Jodele et al. 2020). In secondary TMAs, such as those associated with systemic lupus erythematosus, malignancies, or hematopoietic stem cell transplantation (HSCT), cytokine profiles are often dominated by TNF-α, IL-6, IL-1β, and VEGF, reflecting systemic immune activation. HSCT-associated TMA models suggest that endothelial injury driven by high-dose conditioning regimens, calcineurin inhibitors, and graft-versus-host disease is exacerbated by a cytokine storm-like milieu, sustaining endothelial activation and microvascular thrombosis (Jodele et al. 2020). To aid clarity, a summary table (Table 1) has been included, highlighting key cytokines, their levels in various TMA subtypes, and the model system (human, murine, or in vitro) used for detection. This integrative approach underscores the heterogeneity and commonalities in cytokine-mediated vascular injury and provides a foundation for developing cytokine-targeted therapies and biomarker-guided diagnosis.

### Genetic ultra-rare variants and acquired autoantibodies in complement-mediated TMA

Complement-mediated thrombotic microangiopathy (TMA), particularly atypical hemolytic uremic syndrome (aHUS), is increasingly recognized as a disease driven by both genetic and acquired factors that dysregulate the alternative complement pathway. To enhance clarity and align with current nomenclature, genetic abnormalities are now referred to as “ultra-rare variants” rather than broadly as mutations, reflecting their extremely low frequency in the general population and their significant pathogenic potential. These ultra-rare variants primarily affect genes encoding complement regulatory proteins such as complement factor H (CFH), complement factor I (CFI), membrane cofactor protein (MCP or CD46), complement component C3, and complement factor B (CFB). These variants lead to a loss of function in regulators or gain of function in activating components, resulting in unchecked complement activation on endothelial surfaces, culminating in microvascular thrombosis (Loirat et al. 2016).

Alongside genetic predisposition, acquired autoantibodies play a crucial role in complement dysregulation. Among these, autoantibodies against factor H (anti-FH autoantibodies) are the most clinically relevant and well-characterized. These autoantibodies inhibit factor H function, impairing complement regulation and precipitating endothelial damage and TMA episodes, particularly in children and young adults. While other autoantibodies have been described, their pathogenic significance remains less clear, and anti-FH antibodies remain the primary target of diagnostic and therapeutic interest (Zipfel et al. 2020). By clearly distinguishing these two pathogenic mechanisms—ultra-rare genetic variants and acquired anti-FH autoantibodies—this review focuses on the molecular heterogeneity of complement-mediated TMA and underscores the importance of genetic screening and autoantibody testing for accurate diagnosis and personalized management strategies.

### Cytokine-mediated amplification of immune complex-driven endothelial injury in secondary TMA

In immune-complex mediated diseases such as systemic lupus erythematosus (SLE), cytokines play a pivotal role in exacerbating thrombotic microangiopathy (TMA) by amplifying complement activation and promoting endothelial dysfunction. The deposition of circulating immune complexes on the microvascular endothelium triggers a local inflammatory response, which is significantly influenced by cytokine signaling. Proinflammatory cytokines such as tumor necrosis factor-alpha (TNF-α), interleukin-1β (IL-1β), and interferon-gamma (IFN-γ) contribute to this process by enhancing complement cascade activation, which further damages the endothelial surface and propagates microthrombus formation. Moreover, these cytokines increase vascular permeability and upregulate the expression of endothelial adhesion molecules including ICAM-1 and VCAM-1, facilitating the recruitment and retention of immune cells and promoting immune complex deposition. This cytokine-driven environment not only perpetuates endothelial injury but also sets the stage for a vicious cycle of inflammation, complement activation, and thrombosis, which is central to the pathophysiology of secondary TMA in autoimmune diseases. Therapeutic interventions targeting these cytokine-mediated pathways may therefore hold promise in mitigating vascular injury and improving outcomes in immune complex-associated TMAs (Kitching et al. 2020).

### The two-hit hypothesis in thrombotic microangiopathies

The clinical manifestation of thrombotic microangiopathies (TMAs), particularly complement-mediated forms such as atypical hemolytic uremic syndrome (aHUS), is best explained by the “two-hit” hypothesis. This model proposes that a genetic predisposition alone is insufficient to cause overt disease; rather, a second environmental or physiological insult is required to overwhelm the intrinsic resilience of the vascular endothelium and trigger the pathological cascade leading to TMA. The first hit typically involves ultra-rare genetic variants in complement regulatory genes—such as those encoding factor H, factor I, or membrane cofactor protein—that result in partial loss of function or dysregulation of the alternative complement pathway. These inherited variants confer a susceptibility to excessive complement activation on endothelial surfaces but usually do not cause endothelial injury or thrombosis by themselves. The second hit consists of external triggers that induce endothelial stress or injury and exacerbate complement activation. Common precipitants include infections, pregnancy, certain drugs (e.g., calcineurin inhibitors), autoimmune flares, or transplantation-related factors. This insult disrupts endothelial homeostasis, promotes cytokine release, and tips the balance towards microvascular thrombosis and clinical TMA manifestation. This “two-hit” framework underscores the importance of both genetic screening and careful management of potential triggers in at-risk individuals. It also provides a rationale for therapeutic strategies aimed at reinforcing endothelial protection and controlling complement activation to prevent or mitigate TMA episodes(Zipfel et al. 2020; Kitching et al. 2020).

### Therapeutic strategies: the central role of corticosteroids in immune-mediated TMAs

In the management of thrombotic microangiopathies (TMAs), especially immune-mediated forms such as thrombotic thrombocytopenic purpura (TTP), corticosteroids remain a cornerstone of therapy. Their potent anti-inflammatory and immunosuppressive effects broadly dampen the pathogenic immune responses that drive endothelial injury and microvascular thrombosis. Corticosteroids exert multiple beneficial effects by inhibiting proinflammatory cytokine production, reducing immune complex formation, and suppressing autoantibody synthesis—particularly relevant in TTP where autoantibodies target ADAMTS13, leading to platelet aggregation and microthrombi formation. Clinical evidence supports the use of corticosteroids alongside plasma exchange as first-line treatment to achieve rapid disease control and reduce relapse risk. By contrast, cytokine-specific blockers (e.g., IL-6 inhibitors, TNF-α antagonists) have a more targeted mechanism but remain less established in TMA treatment. While these agents show promise in modulating particular inflammatory pathways implicated in endothelial dysfunction and complement activation, their use is largely investigational or adjunctive. Furthermore, the heterogeneity of cytokine profiles across TMA subtypes complicates the selection of appropriate cytokine-targeted therapies (Loirat et al. 2016; Zipfel et al. 2020; Kitching et al. 2020).

### Therapeutic implications and targeted therapies

The evolving understanding of cytokine-mediated endothelial injury in thrombotic microangiopathies (TMAs) has significant therapeutic implications. While traditional treatment strategies have largely focused on correcting the underlying etiology—such as plasma exchange in thrombotic thrombocytopenic purpura (TTP) or complement inhibition in atypical hemolytic uremic syndrome (aHUS)—emerging data suggest that targeting pro-inflammatory cytokine pathways may serve as a beneficial adjunct or alternative approach, particularly in cases with persistent inflammation or refractory disease (Song et al. 2022; Cai et al. 2023). In TTP, the cornerstone of therapy remains therapeutic plasma exchange, which serves to remove anti-ADAMTS13 autoantibodies and replenish functional ADAMTS13. However, during acute episodes, elevated levels of cytokines like interleukin-6 (IL-6) and tumor necrosis factor-alpha (TNF-α) have been associated with disease severity and delayed recovery. Adjunctive use of corticosteroids is standard to suppress autoimmunity and systemic inflammation, and agents such as rituximab help deplete B-cells responsible for antibody production. The potential use of anti-IL-6 agents, such as tocilizumab, has been explored in small case series, showing promise in reducing inflammation and expediting platelet recovery, though larger studies are needed to confirm efficacy (Hyeon et al. 2025; Evangelidis et al. 2024; Chang et al. 2021).

In aHUS, the advent of eculizumab, a monoclonal antibody against complement component C5, has revolutionized management by effectively halting complement-mediated endothelial damage. Interestingly, eculizumab may also exert indirect anti-inflammatory effects by limiting the generation of pro-inflammatory complement fragments (e.g., C5a), which are known to stimulate cytokine release. In patients with suboptimal response to complement inhibition or in secondary TMAs with overlapping inflammatory features, anti-cytokine therapies could be considered as adjuvants. For example, anakinra, an interleukin-1 receptor antagonist, and TNF inhibitors like infliximab have theoretical benefit, although their use in TMAs remains largely investigational (Mazzierli et al. 2023; Minoia et al. 2021; Thompson and Kavanagh 2022). Secondary TMAs, particularly those associated with autoimmune diseases, transplant, or pregnancy-related conditions, present unique therapeutic challenges. In these contexts, systemic inflammation plays a dominant role in disease propagation. Targeted cytokine blockade may be particularly beneficial here. For instance, elevated IL-6 levels in HELLP syndrome or SLE-associated TMAs suggest a rationale for anti-IL-6 therapy in severe, refractory cases. Additionally, general anti-inflammatory measures, such as corticosteroids, intravenous immunoglobulin (IVIG), and immunosuppressive agents, remain critical in blunting the cytokine storm and controlling the primary disease process (Young et al. 2021; Font et al. 2022; Yang et al. 2022).

## Conclusion

Thrombotic microangiopathies represent a complex group of disorders unified by microvascular thrombosis and endothelial injury, but driven by diverse etiologies. Increasing evidence has placed pro-inflammatory cytokines at the center of this pathophysiological process, functioning not only as mediators of endothelial activation but also as amplifiers of the thrombo-inflammatory cascade that characterizes TMAs. Whether in Shiga toxin-associated HUS, complement-mediated aHUS, autoimmune-related TMA, or thrombotic thrombocytopenic purpura, cytokines such as TNF-α, IL-6, and IL-1β play critical roles in driving endothelial dysfunction, promoting coagulation, and sustaining the inflammatory milieu. This growing understanding of cytokine involvement provides important diagnostic and therapeutic insights. Cytokine profiling may offer prognostic information and help identify patients at risk of severe disease or relapse. More importantly, it supports the rationale for exploring cytokine-targeted therapies—either as adjuncts to standard treatment or as tailored options in specific clinical settings with dominant inflammatory features. Agents such as IL-6 receptor antagonists, TNF inhibitors, and IL-1 blockers represent promising candidates in the evolving therapeutic landscape of TMAs.

## Data Availability

No datasets were generated or analysed during the current study.
